# Knowledge, attitude, and perceived barriers regarding colorectal cancer screening practices and risk factors among medical students in Saudi Arabia

**DOI:** 10.1186/s12909-019-1857-7

**Published:** 2019-11-14

**Authors:** Asma Althobaiti, Hoda Jradi

**Affiliations:** King Abdullah International Medical Research Center, Ministry of National Guard Health Affairs, King Saud Bin Abdulaziz University for Health Sciences, College of Public Health and Health Informatics, Department of Community and Environmental Health, Riyadh, Saudi Arabia

## Abstract

**Background:**

Colorectal cancer (CRC) is a major health problem. It is the third most diagnosed common tumour and the fourth leading cause of cancer-related deaths worldwide. Early screening has been shown to decrease the incidence of CRC cancer and decrease mortality. In Saudi Arabia (SA), there is no national policy for CRC screening despite the growing incidence of the disease. This study investigated the knowledge of risk factors for CRC, recommendations for screening, and attitudes and barriers towards screening among medical students.

**Methods:**

Data was collected using a self-administered valid and reliable questionnaire consisting of demographic characteristics, knowledge, attitude, and barriers measurements completed by 581 medical students from two Saudi schools. Frequencies and mean scores of knowledge and attitude were determined. The likelihood of students having adequate knowledge of CRC risk factors and screening modalities was estimated using multivariate logistic regression analysis.

**Results:**

Knowledge of the risk factors for CRC and screening modalities, and attitude towards screening were poor in 52.47 and 57.83% of the surveyed medical students; respectively. Higher level of medical education (OR = 3.23; 95% CI: 2.01–5.18) and a positive attitude towards CRC screening (OR = 2.74; 95% CI: 1.86–4.03) were independent predictors of higher knowledge levels. Lack of awareness about CRC and screening modalities among patients, and shortage of specialized healthcare providers were barriers independently associated with low knowledge levels.

**Conclusions:**

Saudi medical students have limited knowledge of CRC risk factors of and a poor attitude towards CRC screening. These results contribute to our understanding of missed teaching opportunities in Saudi medical schools and suggest intervening at the medical school, clinical practice, and population levels to increase CRC screening practices.

## Introduction

Colorectal cancer (CRC) is the third most commonly diagnosed tumor and the fourth leading cause of cancer-related death worldwide [1]. By the year 2030, the burden of CRC is estimated to rise by 60% to more than 2.2 million new cases, with an estimated 1.1 million deaths [1]. Almost 55% of cases occur in more developed regions [2]. The most recent report by the Saudi Cancer Registry (2014) cited 1347 cases of colorectal cancer (CRC) in the country; accounting for 11.5% of all newly-diagnosed cases among Saudi nationals [3]. According to this report, CRC affected 753 (55.9%) males and 594 (44.1%) females; thus ranking as the leading cause of cancer in males and the third leading cause of cancer in females [3]. Studies conducted in Saudi Arabia have shown that there was a steady increase in the incidence of CRC from 2000 to 2006 and that the incidence was greater in males than in females [4]. The World Health Organization (WHO) has stated that 40% of cancers could be avoided by prevention and 40% could be cured if detected early [5]. In Saudi Arabia, in addition to the lack of availability of specific resources for the diagnosis of CRC, there is no documentation relating to consistent and organized screening programs at the national level [6]. Moreover, the knowledge of the population is less than that required to encourage the spread of screening behavior [7–9]. Optimal screening strategies for CRC include the annual use of fecal immunochemical testing (FIT), flexible sigmoidoscopy every ten years with an annual FIT test, or a colonoscopy every ten years [10]. These strategies will result in a gradual increase in the number of years with a good quality of life, but will also increase the need for more colonoscopies to be performed at the society level [10].

Age has been established as a main factor underlying the incidence of CRC; the likelihood of colorectal cancer diagnosis increases after the age of 40 and rises sharply after the age of 50 [11]. In addition, several other factors have been implicated as risk factors for CRC, including genetic or familial polyposis, alcohol, smoking and ulcerative colitis [12]. Colorectal cancer prevention strategies involve screening practices in the primary healthcare setting where the early diagnosis of chronic illnesses and cancers is mainly made by primary health care physicians (PHPs) [13]. However, in Saudi Arabia, there is excessive reliance on treatment approaches and a general disregard towards prevention and early detection [13]. Recommended interventions with which to promote prevention and early detection involve enhancement of the present medical education, starting at the academic degree level [14]. Information relating to the deficit in knowledge among medical students could help teachers to develop better teaching strategies and learning outcomes [15]. Medical educators have indicated the importance of cancer prevention in the medical school curriculum because it can result in long-term retained knowledge, positive self-reported attitudes and beliefs, and an intent to apply prevention in future practice [16]. This study, therefore aimed to assess the level of knowledge of CRC risk factors and related screening modalities, attitude towards screening, and barriers regarding CRC screening practices among medical students in Saudi Arabia.

## Methods

### Data collection and sampling

Knowledge of CRC risk factors and screening modalities, attitude towards screening, and barriers associated with screening were investigated in a sample of medical students in years 4 to 6 in two medical schools in the cities of Makkah and Jeddah, Saudi Arabia. The data were collected at the beginning of the school 2018–2019 year (from August to November) using a self-administered questionnaire. The questionnaire was written in English, and all questions were adopted from established measures that have been proven to be valid and reliable for the same purpose in a similar group [16–18]. The clarity of the questions was checked by administering the survey to a group of 10 medical students from different colleges that were not included in the sample. No major modifications were suggested. The reliability of the instrument was checked by administering the questionnaire twice to the same group of students (*n* = 20) with a one-week interval in-between. There was a 98% agreement between the two questionnaires. Sampling was purposeful and included all medical students who gave consent to participate from the designated years of medical education (years 4, 5 and 6) that were present at the time of data collection. Students were recruited from the advanced years of medical education because those in the earlier years are still learning the basic sciences and are less likely to focus on clinical practices. A total of 964 medical students were asked to participate in the study after explaining its purpose, and 581 gave consent to be included in the study (a response rate of 60.27%). All procedures performed in this study were in accordance with the ethical standards of the institutional research committee and with the 1964 Declaration of Helsinki and its later amendments or comparable ethical standards. The study was approved by the Institutional Review Board at King Abdulaziz University for Health Sciences located at King Abdullah International Medical Research Center (Reference: SP 18\066\R).

### The instrument

Demographic characteristics of the students such age, gender, and medical school year were collected. Knowledge relating to colorectal cancer screening tests was assessed by several questions regarding the efficacy of various recommended cancer screening methods (fecal occult blood test, flexible sigmoidoscopy, colonoscopy and double contrast barium enema). This section listed seven screening methods and asked the students to indicate whether they knew if they were very ‘effective’, ‘somewhat effective’, ‘not effective’ or ‘did not know’. The second section contained questions regarding the risk factors for colorectal cancer. This was accomplished by asking students to choose from a list of potential risk factors. The possible range of scores for knowledge was between 0 and 19. The scores were converted into percentages, and the obtained percentage scores were reported in terms of two categories: a high level of knowledge (≥11; a cut-off value of ≥50% correct response score) or a low level of knowledge (< 11; a cut-off value of < 50% correct response). The chosen cut-off value of was based on the mean and median value of approximately 11 for the knowledge score among this group of medical students. The questionnaire also included inquiries related to their personal attitude regarding CRC screening and perceived barriers to patients receiving CRC screening. These items were designed with a stem that included 5 statements (e.g., attitude statement – I would pay for colorectal cancer screening if my health insurance did not cover the cost) and responses ranged from ‘strongly agree’ to ‘strongly disagree’; with a possible range of scores for attitude between 0 and 10. The scores were converted into percentages, and the obtained percentage scores were reported in terms of two categories: a good attitude (≥5) or a poor attitude (< 5) based on the average attitude score of approximately 5 in this population of medical students. The questions regarding patient and health system barriers were approached in the same manner with 12 statements; with 7 of them being patient-related barriers and 5 of them being health system related barriers (e.g., patient barrier – patient fear of finding cancer; system barrier – lack of knowledge about colorectal cancer guidelines). Response choices were ‘major barrier’, ‘minor barrier’ or ‘not a barrier’.

The age of the participants was grouped into three categories: < 22 years, 22–23 years and > 23 years of age. Medical school years were categorized into three classes: 4th, 5th and 6th year.

### Statistical analysis

Analyses were performed using STATA statistical software (version 14; College Station, TX, USA). Descriptive statistics, as means and frequencies, were calculated for all study variables where applicable. Differences in the categories of reported knowledge across study variables (demographic characteristics, attitude and barriers) were assessed. The chi-square test for categorical variables was used to identify significant differences in the parameters.

Multivariate logistic regressions analyses were performed with reported knowledge as the dependent variable to examine the simultaneous effects of the studied factors on the likelihood of high knowledge versus low knowledge. The odds ratios and 95% confidence intervals of a high level of knowledge compared to a low level of knowledge were calculated for a range of study variables. First, all demographic variables that showed significance in the bivariate analysis were entered into the logistic model. Stepwise backward elimination of non-significant variables was then applied (significance level set at *p* < 0.05).

## Results

### Characteristics of the participating medical students

A total of 581 students participated in this study. Detailed demographic characteristics of the study cohort are shown in Table 1. The majority (52.07%) of the students were females and mostly (48.36%) between the ages of 22 and 23 years. More than half (62.48%) of the sample were from Makkah while only 37.52% were from Jeddah. The highest proportion (37.07%) of the sample were in their 4th year, 34.83% were in their 5th year, and 28.10% were in their 6th year.

**Table 1:** Characteristics of the participating medical students (*N* = 581)

Characteristics	N	%
Age		
< 22 years	139	23.92
22–23 years	281	48.36
> 23	161	27.71
Gender		
Male	278	47.93
Female	302	52.07
Medical school year		
4th	215	37.07
5th	202	34.83
6th	163	28.10
College location		
Makkah	363	62.48
Jeddah	218	37.52
Knowledge regarding CRC*(μ =10.98 ± 3.64)		
High	266	45.78
Low	315	54.78
Attitude towards CRC*(μ = 5.23 ± 2.12)		
Good	245	42.17
Poor	336	57.83

*CRC: Colorectal Cancer

### Medical students ‘knowledge and attitude regarding CRC

Most (60.76%) of the medical students agreed that a colonoscopy is a very effective cancer screening method and approximately 30.0% of them claimed that the fecal occult blood test is also a very effective method. Sigmoidoscopy as a screening method was thought to be effective/somewhat effective by 71.95% of this sample of medical students; 41.14% reported that double-contrast barium enema is not effective or did not know about it.

Regarding knowledge of risk factors for colorectal cancer screening, most of the participating medical students reported that family history and an increase in age are risk factors for developing CRC (77.59 and 67.76%; respectively). Also, around 61.10% believed that dietary behavior is a risk factor and 60.96% also mentioned smoking as a risk factor. Male gender, inflammatory bowel disease and a lack of physical activity were also reported as potential risk factors by 48.79, 44.14, and 34.48% of the sample, respectively. Knowledge score (regarding CRC screening methods and risk factors) was lower than average among 54.78% of this sample of medical students.

Personal attitudes regarding cancer screening varied among the medical students. Approximately 87.91% of the students strongly agreed/agreed that the early detection for CRC is favorable and 77.96% of them agreed that undergoing screening provides peace of mind. On the other hand, 56.55% of participants reported an agreement that screening is not beneficial. Slightly more than half (52.07%) of this sample agreed that colonoscopy is embarrassing. The majority of the students (77.92%) expressed a positive attitude towards paying for CRC screening themselves if not covered by health insurance. Attitude score regarding screening for CRC was poor among 57.83% of participating medical students (results for this section are presented in Table 2).

**Table 2:** Medical student-reported knowledge regarding the effectiveness of screening methods and risk factors for CRC* (*N* = 581)

Knowledge variable (μknowledge score = 10.98 ± 3.64)			
Effectiveness of screening	Very effective	Somewhat effective	Not effective\don’t know
	N (%)	N (%)	N (%)
Fecal Occult Blood test	174 (29.95)	233 (40.10)	174 (29.95)
Colonoscopy	353 (60.76)	149 (25.65)	79 (13.60)
Sigmoidoscopy	211 (36.32)	207 (35.63)	163 (28.06)
Double-contrast barium enema	149 (25.65)	193 (33.22)	239 (41.14)
Risk factor	Yes	No	–
	N (%)	N (%)	
Family history	450 (77.59)	130 (22.41)	–
IBD**	256 (44.14)	324 (55.86)	–
Lack of physical activity	200 (34.48)	380 (65.52)	–
Increasing age	393 (67.76)	187 (32.24)	–
Smoking	352 (60.96)	228 (39.31)	–
Dietary intake	355 (61.10)	226 (38.90)	
Male gender	283 (48.79)	297 (51.21)	–
Attitude variable (μattitude score = 5.23 ± 2.12)			
	Strongly agree	Agree	Disagree/strongly disagree
	N (%)	N (%)	N (%)
Early detection is better	366 (63.21)	143 (24.70)	70 (12.09)
Colonoscopy is embarrassing	148 (25.52)	154 (26.55)	278 (47.93)
Screening gives peace of mind	210 (36.14)	243 (41.82)	128 (22.03)
Willingness to pay for screening	168 (28.92)	239 (41.14)	174 (29.95)
Screening is not beneficial	137 (23.62)	191 (32.93)	252 (43.45)

**CRC, Colorectal Cancer; *IBD: Inflammatory Bowel Disease

### Medical students’ reported barriers regarding CRC screening

The medical students reported patient-related barriers and health system-related barriers to CRC screening. Among the reported patient-related major barriers were a fear of finding cancer (56.72%), experiencing embarrassment or anxiety (53.10%), not knowing about the disease in general (52.76%), and being asymptomatic (51.21%).

The reported health system-related major barriers regarding CRC screening were a lack of knowledge about screening guidelines (57.76%), high cost and lack of coverage (56.38%), lack of recommendations in primary healthcare (45.0%), and a shortage of healthcare providers to conduct screening other than the fecal occult blood testing (42.24%). Results for reported barriers are shown in Table 3.

**Table 3:** Medical student-reported barriers related to patients and healthcare systems, with regards to CRC risk factors and screening (*N* = 581)

Barrier statement	Major Barrier N (%)	Minor Barrier N (%)	Not a Barrier N (%)
Patient-related			
Fear of finding cancer	329 (56.72)	186 (32.07)	65 (11.21)
Belief that screening is not effective	230 (39.66)	244 (42.07)	106 (18.28)
Embarrassment /anxiety	308 (53.10)	194 (33.45)	78 (13.45)
Colorectal cancer not a serious health threat	225 (38.79)	224 (38.62)	131 (22.58)
No symptoms of a health problem	297 (51.21)	176 (30.34)	107 (18.45)
lack of knowledge about colorectal cancer	306 (52.76)	209 (36.03)	65 (11.20)
Procrastination of the procedure	265 (45.69)	240 (41.38)	75 (12.93)
Healthcare system-related			
High cost and lack of coverage	327 (56.38)	184 (31.72)	69 (11.90)
No available recommendations in Primary Healthcare	261 (45.00)	224 (38.62)	95 (16.38)
Shortage of trained providers to conduct screening other than fecal occult blood testing	245 (42.24)	240 (41.38)	95 (16.38)
Shortage of trained providers to conduct follow-up with invasive endoscopic procedures	239 (41.21)	268 (46.21)	73 (12.59)
Lack of knowledge about colorectal cancer guidelines	335 (57.76)	163 (28.10)	82 (14.14)

### Factors associated with knowledge about colorectal cancer and related screening procedures

Bivariate analysis showed that the level of knowledge related to CRC risk factors and screening among this group of medical students varied significantly by age (*p* < 0.001), medical school year (*p* < 0.001), surveyed college (*p* < 0.001) and attitude towards CRC (*p* < 0.001). There were many reported barriers to CRC screening in the participating medical students (Table 4), including patient fear of finding cancer (*p* = 0.018), patient embarrassment or anxiety related to screening (*p* < 0.001), not perceiving CRC as a serious health threat (*p* = 0.005), a lack of symptoms for CRC as a reason not to conduct screening (*p* < 0.001), a lack of knowledge about the disease in general (*p* < 0.001), patients procrastinating about the procedure (*p* = 0.004), high cost or no availability of screening (*p = *0.005), a lack of recommendations for screening in primary care (*p* < 0.001), and a shortage of healthcare providers who can conduct screening (p < 0.001) or follow-up with invasive endoscopic procedure (*p* < 0.001) (Table 4).

**Table 4:** Factors associated with the level of knowledge regarding CRC risk factors and screening among participating medical students (*N* = 581)

Knowledge			
	Low N (%) 315	High N (%) 266	*P*-value
Age			< 0.001
< 22	88 (27.93)	51 (19.17)	
22–23	123 (39.04)	158 (59.39)	
> 23	104 (33.01)	57 (21.42)	
Gender			0.403
Male	156 (49.52)	122 (45.86)	
Female	159 (50.47)	143 (54.13)	
Medical school year			< 0.001
4th	146 (46.34)	69 (25.93)	
5th	97 (30.79)	105 (39.47)	
6th	72 (22.85)	91 (34.21)	
College			< 0.001
College1 (Makkah)	221 (70.15)	142 (53.38)	
College2 (Jeddah)	94 (29.84)	124 (46.61)	
Attitude towards CRC			< 0.001
Good	94 (29.84)	151 (56.76)	
Poor	221 (70.15)	115 (43.23)	
Reported barriers			
Fear of finding cancer	163 (51.74)	166 (62.40)	0.018
Belief that screening is not effective	114 (36.19)	116 (43.60)	0.069
Embarrassment or anxiety about screening tests	141 (44.76)	167 (62.78)	< 0.001
Do not perceive CRC as a serious health threat	102 (32.38)	123 (46.24)	0.005
Do not show any symptoms of a health problem	144 (45.71)	153 (57.51)	< 0.001
Lack of knowledge about CRC	128 (40.63)	178 (66.91)	< 0.001
Procrastinating the procedure	140 (44.44)	125 (46.99)	0.004
Screening costs too much or insurance doesn’t cover	171 (54.28)	156 (58.64)	0.005
No recommendations for screening in primary care	113 (35.87)	148 (55.63)	< 0.001
Shortage of trained providers to conduct screening other than fecal occult blood testing	113 (35.87)	132 (49.62)	< 0.001
Shortage of trained providers to conduct follow-up with invasive endoscopic procedures	99 (31.42)	140 (52.63)	< 0.001
Lack of knowledge about CRC guidelines	176 (55.87)	159 (59.77)	0.501

Further analysis using univariate and multivariate logistic regression, to ascertain the effect of study variables on the level of knowledge about CRC among this sample of medical students was used. The final multivariate logistic regression, showed that being a student in year 5 (OR = 2.97, 95%CI: 1.90–4.65) or year 6 (OR = 3.23, 95%CI: 2.01–5.18), and reporting a good attitude towards CRC and related screening procedures (OR = 2.74, 95%CI:1.86–4.03), were independently associated with a higher level of knowledge. In addition, reporting patient-related barriers to screening such as not perceiving CRC screening as a serious health threat (OR = 0.74, 95%CI:0.58–0.94), asymptomatic CRC (OR = 0.71, 95%CI:0.55–0.91) and patient lack of knowledge about CRC (OR = 0.53, 95%CI:0.40–0.71) were all independently associated with a lower level of knowledge among medical students. The shortage of trained providers to conduct follow-up with invasive endoscopic procedures (OR = 0.58, 95%CI: 0.44–0.78) as a healthcare system-related barrier was also significantly associated with a lower level of knowledge about CRC risk factors and screening procedures (Tables 5 and 6).

**Table 5:** Results of univariate (unadjusted) analysis for factors associated with a high level of knowledge regrading CRC risk factors and screening among medical students (*N* = 581)

Variable	OR	95% CI	P-Value
Participant characteristics			
Age			
< 22 (Ref)*	1.00		
22–23	2.21	1.45–3.36	< 0.001
> 23	0.94	0.59–1.52	0.871
Medical school year			
4th (Ref)*	1.00		
5th	2.29	1.54–3.40	< 0.001
6th	2.67	1.75–4.07	< 0.001
Gender			
Male (Ref)*	1.00		
Female	1.15	0.82–1.59	0.403
College			
College1 (Ref)*	1.00		
College2	2.05	1..45–2.88	< 0.001
Attitude towards CRC **			
Poor (Ref) *	1.00		< 0.001
Good	3.087	2.19–4.35	
Reported Barriers (Ref: barrier non-existent)			
Patient fear of finding cancer	0.70	0.55–0.898	0.005
Patient believes screening is not effective	0.90	0.717–1.122	0.34
Patient embarrassment/anxiety of screening	0.49	0.382–0.630	0.001
Patient does not perceive CRC** as a serious health threat	0.73	0.588–0.906	0.004
Patient has no symptoms of a health problem	0.58	0.471–0.737	< 0.001
Patient lack of knowledge about colorectal cancer	0.44	0.339–0.576	< 0.001
Patient procrastinates the procedure	0.78	0.619–0.991	0.047
Screening costs too much or insurance doesn’t cover	0.75	0.596–0.961	0.023
Lack of recommendations for screening in primary care	0.60	0.480–0.765	< 0.001
Shortage of trained providers to conduct screening other than FOBT***	0.61	0.485–.0777	< 0.001
Shortage of trained providers to conduct follow-up with invasive endoscopic procedures	0.47	0.367–0.618	< 0.001
Lack of knowledge about colorectal cancer guidelines	0.90	0.721–1.12	0.371

*Ref Reference category, **CRC Colorectal Cancer, *FOBT* Fecal Occult Blood Test

**Table 6:** Results of multivariate analysis for factors significantly associated with a high level of knowledge regarding CRC risk factors and screening among medical students (*N* = 581)

Variable	OR	95% CI	P-Value
Education year			
4th (Ref)*	1.00		
5th	2.97	1.90–4.65	< 0.001
6th	3.23	2.01–5.18	< 0.001
Attitude towards CRC **			
Poor (Ref)	1.00		< 0.001
Good	2.74	1.86–4.03	
Reported barriers			
Patient does not perceive CRC** as a serious health threat	0.74	0.58–0.94	0.016
Patient does not show any symptoms of a health problem	0.71	0.55–0.91	0.008
Patient lack of knowledge about CRC**	0.53	0.40–0.71	< 0.001
Shortage of trained providers to conduct follow-up with invasive endoscopic procedures	0.58	0.44–0.78	< 0.001

*Ref Reference category, **CRC Colorectal Cancer

## Discussion

Knowledge regarding colorectal cancer risk factors, screening modalities and attitude towards screening were low among more than half of the surveyed medical students from the two medical schools in Saudi. An advanced number of years in medical school education, and a good attitude towards colorectal cancer screening, were shown to be independent predictors of a higher level of knowledge. Also, a lack of awareness about colorectal cancer and screening modalities among patients, and a shortage of specialized healthcare providers, were reported barriers that were independently associated with a low level of knowledge among this sample of students. The perceived competence among medical students regarding colorectal cancer screening knowledge and practices has been discussed in several countries [16–19], but to the best of our knowledge, not in Saudi Arabia. Previous literature has shown that knowledge about colorectal cancer screening among medical students is inadequate [19]; as evident in the present study. It is common knowledge that awareness about a disease and its prevention is a positive approach towards disease control and prevention. Despite CRC screening leading to a reduction in mortality, colorectal cancer is the third most common malignancy in the world [20]. Educational barriers and behavioral patterns have been acknowledged as major reasons for the low prevalence of screening [21]. It has also been established that knowledge plays an important role in deciding preventive behaviors in any population. To improve colorectal cancer screening rates, trainee physicians must be educated about the relevant tests and their appropriate application. Medical students, as future physicians, should have adequate knowledge about screening practices and guidelines for colorectal cancer in order to develop skills and adopt appropriate guidelines early in their careers; this will help to contain the disease and minimize its burden. The grade of knowledge and competency regarding CRC among medical students, who represent our future physicians, produces necessary implications for their patients [15]. A better understanding of how a medical student’s knowledge and attitudes towards colorectal cancer screening relates to clinical performance would guide educators in developing effective curricula. It has been documented in the literature that CRC prevention strategies involve the enhancement of existing medical education, beginning at the level of the academic degree [14] and that collecting information about knowledge deficit from medical students could help teachers to improve teaching strategies and prepare a more productive curriculum to improve student learning outcomes [15]. Knowledge related to CRC screening methods increased progressively through successive academic years among medical students from both schools involved in our present study; similar findings have been documented previously [16].

Most of our students considered colonoscopy as the most effective cancer screening method, followed by flexible sigmoidoscopy. Only one-third of our students thought that the FOBT was very effective; this is consistent with a study conducted on physicians in Riyadh [13]. Actually, colonoscopy is now the most frequently (95%) recommended test by primary healthcare physicians to asymptomatic, average-risk individuals, followed by FOBT (80%) [22, 23]. Most primary healthcare physicians do not recommend the full list of test options prescribed in the national guidelines [22].

In contrast to the findings of a previous study [17], the majority of our students reported correctly that age is a risk factor in developing colorectal cancer; previously published data found that the incidence rate is more than 50 times higher in persons aged 60 to 79 years compared to those younger than 40 years [2]. In addition, in the present study, approximately three quarters of students knew that CRC family history was a risk factor for CRC and less than half answered correctly that inflammatory bowel disease was associated with CRC. Furthermore, the high proportion of medical students who reported that diet is a risk factor were definitely aware that CRC is a preventable disease via the modification of associated risk factors such as an excessive intake of meat [24]. However, a high proportion of the students underestimated the fact that physical inactivity can be linked to colorectal cancer; epidemiological evidence consistently shows that physical activity reduces the risk of colon cancer [11, 12, 24, 25].

One of the interesting results observed in the current study is that students reported the barrier that a majority of patients are either too embarrassed and/or are afraid of being diagnosed with colon cancer. Also, other reported barriers, such as a lack of patient awareness about CRC and related screening methods, and a shortage of trained healthcare providers, were associated with the level of knowledge among medical students. A lack of awareness and inadequate knowledge and information about CRC, and not perceiving colorectal cancer as a serious health problem, were previously reported in the literature as barriers to screening [26]. In view of this, it could be helpful to identify what attitudes, beliefs, and perceived barriers the students have in order to develop an effective curriculum and training strategy that would assist the students and the population at large in increasing their knowledge about CRC and CRC screening. Regardless, of their knowledge or attitudes, patients might not be able to get recommended screening tests or act upon results if there was a lack of essential resources. In our survey, more than half of the students reported that common barriers to CRC screening were the high cost of tests and inadequate health insurance coverage.

A physician’s knowledge regarding CRC screening has been reported as being acceptable in previously studies conducted in Saudi Arabia; especially among board-certified physicians [13]. This type of information highlights the physician’s role in educating other physicians and students in the importance of the screening. Despite the reported effectiveness of the CRC screening in reducing mortality [14], published findings on CRC practices in Saudi Arabia confirm that practice is currently inadequate [10, 13]. Despite an increase in the incidence of CRC in the country, the Ministry of Health (MOH) does not highlight the role of education at all levels with regards to CRC as compared to other cancers such as breast and lung cancer; currently there is no national screening program for CRC. Therefore, and as suggested by other publications [7, 13, 15], it is fundamental that we motivate the role of health promotion and enrich the level of awareness of CRC in the Saudi population and include CRC education and screening modalities in the curriculum of medical schools as early as possible. Installing the importance of preventive behavior and screening early in the medical career is highly beneficial in terms of disease prevention in any country.

A limitation of this study is that it was carried out in two medical schools in two cities of Saudi Arabia and does not necessarily represent all medical schools in the area or the country. A more important limitation is that we did not collect any information about the actual content of the medical curriculum and whether the students have been exposed to educational material and training regarding CRC, in either the classroom or the clinical setting; these factors could clearly influence their level of proficiency. We cannot exclude the fact that limited knowledge about CRC among students was due to the fact that some of them were in their fourth year of medical school and had experienced limited exposure to clinical training. One additional limitation is that the results of this study are based on cross-sectional data so causal inferences cannot be established.

## Conclusions

The Saudi medical students surveyed in this study reported limited knowledge about CRC screening methodology and many exhibited a poor attitude towards CRC screening. Our study highlights the fact that, as previously observed in some other countries, Saudi medical students do not receive adequate and early training in CRC screening. As a result, medical students may not possess the ability to help in increasing the uptake of CRC prevention by screening and may not be fully aware of the important role that a physician may have in this effort. There is clearly a need to develop uniform curricular requirements for factual knowledge and practical skills regarding CRC screening in medical schools. Future research can be directed towards investigating the content of the medical school curriculum relating to screening for cancer in general and for CRC in particular. In addition, it is important to investigate the current practices in primary healthcare for CRC screening by establishing current screening rates that will help highlight the magnitude of the problem and motivate physicians to spread the knowledge and the practice of screening for CRC among those who are mostly at risk.

## Data Availability

The datasets used and analyzed in this study are available from the corresponding author upon request.

## Abbreviations

CRC: Colorectal Cancer
FIT: Fecal Immunochemical Testing
FOBT: Fecal Occult Blood Test
WHO: World Health Organization
